# Stormtime substorm onsets: occurrence and flow channel triggering

**DOI:** 10.1186/s40623-018-0857-x

**Published:** 2018-05-15

**Authors:** Larry R. Lyons, Ying Zou, Yukitoshi Nishimura, Bea Gallardo-Lacourt, Vassilis Angelopulos, Eric F. Donovan

**Affiliations:** 1 Department of Atmospheric and Oceanic Sciences, University of California, Los Angeles, CA 90095-1565 USA; 2 Center for Space Physics and Department of Astronomy, Boston University, Boston, MA 02215 USA; 3 Cooperative Programs for the Advancement of Earth System Science, University Corporation for Atmospheric Research, Boulder, CO USA; 4 Center for Space Physics and Department of Electrical and Computer Engineering, Boston University, Boston, MA 02215 USA; 5 Department of Physics and Astronomy, University of Calgary, 2500 University Drive, Calgary, AB T2N 1N4 Canada; 6 Department of Earth, Planetary, and Space Sciences, University of California, Los Angeles, CA 90095-1567 USA

**Keywords:** Substorms, Storms, Auroral streamers, Substorm triggering, Substorm occurrence

## Abstract

Bright auroral emissions during geomagnetic storms provide a good opportunity for testing the proposal that substorm onset is frequently triggered by plasma sheet flow bursts that are manifested in the ionosphere as auroral streamers. We have used the broad coverage of the ionospheric mapping of the plasma sheet offered by the high-resolution THEMIS all-sky-imagers (ASIs) and chose the main phases of 9 coronal mass ejection (CME) related and 9 high-speed stream (HSS)-related geomagnetic storms, and identified substorm auroral onsets defined as brightening followed by poleward expansion. We found a detectable streamer heading to near the substorm onset location for all 60 onsets that we identified and were observed well by the ASIs. This indicates that substorm onsets are very often triggered by the intrusion of plasma with lower entropy than the surrounding plasma to the onset region, with the caveat that the ASIs do not give a direct measure of the intruding plasma. The majority of the triggering streamers are “tilted streamers,” which extend eastward as their eastern tip tilts equatorward to near the substorm onset location. Fourteen of the 60 cases were identified as “Harang streamers,” where the streamer discernibly turns toward the west poleward of reaching to near the onset latitude, indicating flow around the Harang reversal. Using the ASI observations, we observed substantially less substorm onsets for CME storms than for HSS storms, a result in disagreement with a recent finding of approximately equal substorm occurrences. We suggest that this difference is a result of strong non-substorm streamers that give substorm-like signatures in ground magnetic field observations but are not substorms based on their auroral signature. Our results from CME storms with steady, strong southward IMF are not consistent with the ~ 2–4 h repetition of substorms that has been suggested for moderate to strong southward IMF conditions. Instead, our results indicate substantially lower substorm occurrence during such steady driving conditions. Our results also show the much more frequent occurrence of substorms during HSS period, which is likely due to the highly fluctuating IMF.

**Electronic supplementary material:**

The online version of this article (10.1186/s40623-018-0857-x) contains supplementary material, which is available to authorized users.

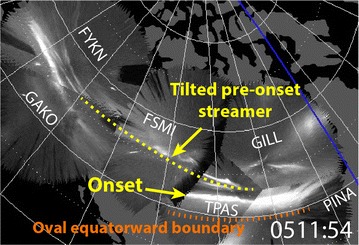

## Introduction

Akasofu (1964) introduced the concept of a substorm using ground-based all-sky imager observations of aurora (Akasofu 1964) that showed a sudden brightening of a quiet arc seen near the equatorward boundary of the auroral oval that subsequently expanded poleward. The arc that brightens identifies the onset of the substorm expansion phase, and its mapping along magnetic field lines is likely to the near-Earth portion of the electron plasma sheet (Samson et al. 1992). As a major disturbance of the magnetosphere-ionosphere system, substorms have been studied extensively from the ground and in space. However, the sequence of events leading to substorm onset was elusive and remained a subject of intensive debate for decades. The main debate had been whether substorm onset is triggered by the onset magnetic reconnection in the mid-tail plasma sheet (~ 20–30 *R*_*E*_, downtail from the Earth) or by a process that disrupts current along near-Earth plasma sheet field lines (~ 10 *R*_*E*_ downtail) (Angelopoulos 2008).

A resolution to this long-standing problem was proposed by Nishimura et al. (2010a) using observations from the Time History of Events and Macroscale Interactions during Substorms (THEMIS) all-sky-imager (ASI) array. These imagers provided the first high temporal and spatial resolution auroral images with broad longitudinal and latitudinal coverage (Mende et al. 2008), allowing detection of weak auroral forms, as well as auroral features over a broad spatial scale relative to the more localized strong auroral brightening of substorm onset. They found evidence that the pre-onset auroral sequence is different from what has been generally considered previously, being initiated by an intensification along the poleward boundary of the auroral oval (a PBI) that is followed by a discrete auroral form that traverses magnetic latitude lines to lower latitudes. Such auroral forms are now commonly referred to as an auroral streamer [referred to as a N–S arc by Nishimura et al. (2010a)] that extends equatorward to the vicinity of the location of auroral onset. While such triggering had been previously noted from much lower quality imaging (Oguti 1973), it was not viewed by the community as a common phenomenon that had important implications for the magnetosphere-ionosphere coupling that lead to substorm onset. Nishimura et al. (2010a) altered the debate toward consideration of the extent to which this new streamer-triggering scenario was correct and physical processes implied by this triggering (e.g., Nishimura et al. 2010b; Frey 2010; Nishimura et al. 2013; Rae et al. 2013). In the present paper, we evaluate streamer triggering by taking advantage of the brighter than normal auroral emissions that occur during geomagnetic storms, thus facilitating substorm onset and streamer identification. Based on our identified substorm onsets, we also determine substorm onset occurrence rates during the main phases of coronal mass ejection (CME) and high-speed solar wind stream (HSS) storms.

The new streamer-triggering scenario, if generally true, would have important implications for understanding the physical processes that lead to substorm onset. Streamers have been definitively related to narrow flow channels in the ionosphere (Gallardo-Lacourt et al. 2014b). These ionospheric flows are the mapping of enhanced earthward flows within the plasma sheet that have been related to streamers (Rostoker et al. 1987; Sergeev et al. 1999, 2000; Zesta et al. 2000; Henderson et al. 2002; Pitkänen et al. 2011; Haerendel 2011). Consistent with what is seen in the ionosphere, the plasma sheet flows are localized in the cross-tail direction (Angelopoulos et al. 1996; Sergeev et al. 1996; Nakamura et al. 2004), so that they can be viewed as channels or bursts of earthward flowing plasma. It was proposed that these plasma sheet flow bursts consist of depleted magnetic flux tubes (i.e., flux tubes with lower total entropy than the surroundings, leading to earthward interchange motion) (e.g., Pontius and Wolf 1990; Yang et al. 2011; Wolf et al. 2012), an idea that now has considerable support from observations (Dubyagin et al. 2010; Panov et al. 2010; Sergeev et al. 1996, 2012; Xing et al. 2010).

While the low-entropy plasma is presumable brought earthward by interchange motion, onset appears to result from a separate instability that is triggered by the intrusion of new, reduced entropy plasma to near the inner edge of the electron plasma sheet. Auroral observations have also revealed important features of this instability, including that the initial brightening occurs along a preexisting arc (which may have recently formed). The brightening is often observed to start with a wavy pattern, which is referred to as auroral beads (Donovan et al. 2006; Sakaguchi et al. 2009). These waves grow in amplitude and are accompanied by the rapid development of strong electric fields with each auroral bead (Gallardo-Lacourt et al. 2014a).

It needs to be emphasized that it is not the auroral streamers by themselves that are important in this proposed scenario. Rather, it is the lower entropy plasma that is brought into the inner portion of the plasma sheet. The reduced entropy flow channels are narrow so that they are difficult to see from observations by sparse spacecraft. However, continent-scale ground-based auroral observations offer the opportunity for broad coverage of the ionospheric mapping of the plasma sheet. They can be used both for the detection of substorm onsets and for pre-onset streamers.

In the following, we first describe our event selection and then evaluate the frequency of occurrence of detected substorm onsets separately for CME storm main-phase periods and HSS storm main-phase periods. We believe it is interesting to see how our results using high-resolution auroral observations compare with previous results that did not directly use such observations. In particular, we compare to the idea based partially on particle injections that substorms might repeat with an ~ 2–4 h period (referred to as “sawtooth events”) during storms that are driven by moderate to strong (Bz ≲ − 10 nT) and continuously southward IMF conditions (Henderson et al. 2006, and references therein) and to the recent evaluation indicating that substorm occurrence rates might be similar during CME and HSS storms (Liou et al. 2017). We also compare to apparently conflicting proposals. The first is that HSSs, which contain high amplitude Alfvén waves in the interplanetary magnetic fields (IMF), are associated with prolonged periods of geomagnetic activity (Tsurutani and Gonzalez 1987) that includes numerous substorms (Kim et al. 2008). The second is evidence that storms with smooth IMF variations show a very low occurrence of substorms (Tsurutani et al. 2003). Finally, we evaluate the rate of apparent streaming triggering of substorms for onsets that are not obscured by clouds, the moon, or trees and having initial brightening detected within the field-of-view (FOV) of the ASI imagers.

## Event selection and substorm onset occurrence rates

**Fig. 1:**
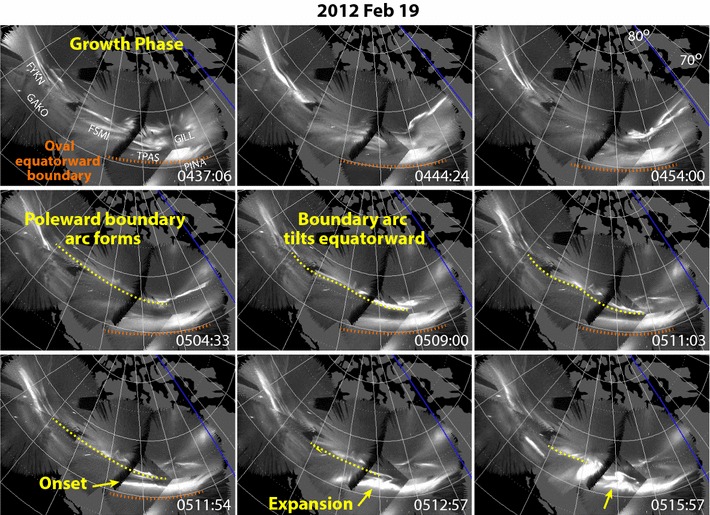
Representative mosaics of images on 19 February 2012 from the THEMIS ASIs over North America showing the motion of a tilted streamer as it extended to a substorm onset in the equatorward portion of the auroral oval. UT is each mosaic is given in the lower right of each panel, longitude lines are separated by 1 h in MLT, and ASI stations used in the mosaics are identified in the upper left panel

We have selected 9 storm days driven by coronal mass injections (CMEs) and 9 storm days driven by high-speed solar wind streams (HSSs) having main phases activity over North America and having good viewing over North America with the THEMIS ASI array. Following the classical Akasofu (1964) definition, we first identified all substorm onsets seen by the ASIs via a brightening of an east–west oriented auroral arc within the equatorward portion of the auroral oval that is followed by poleward expansion of the auroral activity (brightenings without poleward expansion were not included, though these were few in number). We are interested in what leads to onset, and not the following substorm development. Thus, both full substorm onsets and pseudo-breakups (where auroral activity starts to expand poleward but does not reach the auroral poleward boundary) were included, and we did not impose a minimum time separation between onsets. We only required that previous substorm activity fades before a new substorm onset was identified. An example of an onset identified at 0511:21 with the ASI array on 19 February 2012 is shown in Fig. 1 and can be seen in the movie Additional file 1, which shows images every 3 s.

All selected storm days and identified onset times are given in Table 1. Pseudo-breakups are identified by a maroon p followed by 1, 2, or, 3, which give the observed degrees of latitude of pseudo-breakup poleward expansion estimated from the images. The table is divided between CME and HSS storms. Only 10 of 80 total onsets were pseudo-breakups, with no evident difference between CME and HSS storms. It is interesting that 50% more substorm onsets were identified during HSS storms than during CME storms. This suggests that the smoothness of the IMF during CME relative to that during HSS storms may be an important factor in the substorm onset occurrence difference seen in Table 1, a result more consistent with the proposals of Tsurutani and Gonzalez (1987), Kim et al. (2008), and Tsurutani et al. (2003) than with the results considered in Henderson et al. (2006) and Liou et al. (2017).

**Table 1:** Selected storm days and identified onsets times and onset attributes sorted by CME and HSS storms

CME storm date	# substorms	# obs + # FOV	# HA	Substorm UTs in ASIs
2012 Feb 19	5		1	0253p2 (TS) 0305 (TS), 0328 (HA), 0402 (TS), *0511 (TS)*
2012 Oct 1	3			0125 (TS), 0202 (TS), 0421 (TS)
2012 Oct 9	5	4	1	0217 (obs), 0239 (obs), 0700 (HA), 0942 (FOV), 1014 (obs)
2012 Oct 13	4	1		0739p3 (TS), 0744p3 (TS), 0803 (TS), 1113 (FOV)
2014 Feb 19	3	2		0731 (TS), 0852p3 (FOV), 0903 (FOV)
2014 Feb 20	6	1	2	0505 (TS), 0634p1 (HA), 0641 (HA), 0703 (TS), 0759 (TS), 1015 (FOV)
2014 Feb 28	2			0310 (TS), 0749 (TS)
2014 Apr 12	4	2		0640 (TS), 0713 (TS), 0931 (obs), 1025 (obs)
2014 Apr 30	0			
Totals	32	10	4	

HSS storm date	# substorms	# obs + # FOV	# HA	Substorm UTs in ASIs
2006 Jan 26	5	2		0446 (TS)p2, 0643 (obs), 0837 (TS), 0856 (TS), 1242 (FOV)
2006 Sep 24	9	2	1	0213 (TS), 0328 (HA), 0406 (TS), 0507 (TS), 0546 (FOV), 0605 (TS), 0628p2 (TS), 0922 (TS), 1029 (FOV)
2006 Nov 10	4	1	1	0358 (FOV), 0736 (TS), 0807 (HA), 0932 (TS)
2008 Mar 09	4		2	0143 (TS), 0529p3 (HA), 0648 (TS), 0721 (HA)
2008 Mar 27	6		1 (+ 1?)	0218 (TS), 0539 (HA), 0637p3 (TD/HA?), 0700 (TS), 0728 (TS), 0827 (TS)
2008 Mar 28	5		1	0327 (TS), 0600 (TS), 0623 (TS), 0824 (HA), 0832 (TS)
2008 Sep 04	5	2	1	0413 (TS), 0436 (FOV), 0614 (TS), 0650 (FOV), 0725 (HA)
2011 Mar 02	5	2	2	0026 (FOV), 0315 (TS), 0520p2 (HA), 0624 (HA), 0654 (FOV)
2015 Mar 2	5	1	1	0546 (HA), 0631 (TS), 0709 (TS), 0716 (TS), 1057 (obs)
Totals	48	10	10(+ 1?)	

Pseudo-breakups are identified by a maroon p followed by 1, 2, or 3, which give the observed degrees of latitude of pseudo-breakup poleward expansion. The attributes are TS (tilted streamer); HA (Harang streamer); Ob: (onset obscured by moon, clouds, or trees); FOV (onset detected by initial brightening slightly beyond ASI FOVs)

**Fig. 2:**
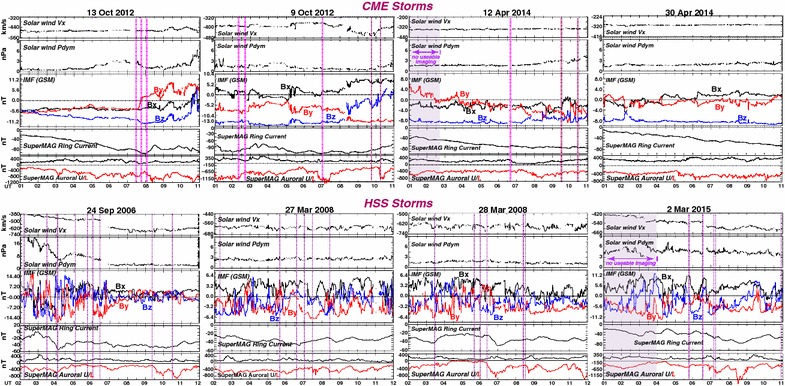
Solar wind and geomagnetic parameters for the four of our selected CME events having the smoothest southward IMF Bz during the period of ASI viewing. The same parameters are also shown for four HHS storms with fluctuating IMF during their period of ASI viewing. For each storm, vertical dashed lines give all the substorm onsets identified with the ASI array. Magenta dashed line identify the 7 substorm identified during steady, strong southward IMF of the CME storms. Pdym is solar wind dynamic pressure. The SuperMAG ring and auroral U and L indices are the same as the standard ring current Sym-H and auroral AU and AL indices, except from a large array of available magnetometer stations

To investigate the possible influence of the smoothness of the IMF, Fig. 2 shows solar wind and geomagnetic parameters for the four of our selected CME events having the smoothest southward IMF Bz during the period of ASI viewing. The same parameters are also shown for the four HSS storms having the best combination of good ASI viewing and fluctuating IMF during the 10 h period shown. As examples of the aurora seen during these storms, Additional file 2 shows images every 1 min for 02-11 UT for the CME storm on 30 April 2014, and Additional file 3 shows images every 1 min for 02-11 UT for the 27 April 2008 HSS storm. For each storm, vertical dashed lines in Fig. 2 give all the substorm onsets identified with the ASI array. During the CME storms, only 7 substorm onsets (magenta dashed lines) were identified during 31.5 h of steady, strong southward IMF with useable ASI viewing, or 0.2 onsets/h. (Only 3 of those, at 0220 UT and 07 UT on 9 Oct and 0640 UT on 12 April, were also during relatively steady IMF By), On the other hand, 25 substorm onsets were identified during the 33 h of useable viewing during the HSS storms, or 0.8 onsets/h. This is quite a remarkable difference, identified substorm onsets being ~ 4 times more frequent during fluctuating IMF than during steady strong southward IMF conditions, despite the steady IMF having substantially strong southward IMF. We note that solar wind dynamic pressure was quite stable during most of the onsets for both the CME and HSS storms, indicating that the difference in most likely related to the steadiness/fluctuations of the IMF. Also, many of the onsets do not coincide with a sharp drop in AL (the SuperMAG AL index, with many more stations than the normal AL, is shown), this likely being due to strong currents associated with the generally active conditions that accompany a storm giving a mostly continuous strong depression of AL.

## Evaluation of streamer triggering of onset

Our determination of whether a given substorm onset is consistent with the Nishimura et al. (2010a) scenario is based on whether a streamer is detected approaching the substorm onset location during a period of time ending at the first detection of onset. Since it is the incoming channel of low-entropy plasma adjacent to the streamer that is critical for the onset, and the flow channels are typically ~ 100 km in width (Gallardo-Lacourt et al. 2014b), the streamer is expected to reach near (within ~ 1°) of the onset latitude. Also, the streamer can be a little to the east of the actual onset due to the westward magnetic drift of low-entropy plasma when it approaches the growth phase arc as seen in Rice Convection Model simulations (Yang et al. 2014). We do not consider the 20 of 80 identified substorm onsets where the initial onset brightening was either obscured (by clouds, trees, or moon) or was just outside the FOV of the imagers.

The example in Fig. 1 and movie Additional file 1 shows a clear example of the type streamer triggering that we commonly see. In this case, the oval was a few degrees in latitude thick and had activity along and near the auroral poleward boundary during the substorm growth phase. The equatorward boundary of oval is identified in each image prior to and at onset via the equatorward boundary of a band of diffuse emissions, this boundary moving equatorward during the growth phase. An arc can be seen to have formed along the auroral poleward boundary (identified in the 0504:33 UT panel of Fig. 1) ~ 7 min before onset, extending across ~ 4 h of MLT. The arc was not strictly east–west, tilting equatorward from west to east. This equatorward tilt then increased with time, the eastern portion of the arc moving equatorward within the oval and approaching the oval equatorward boundary. We call such tilted arcs “tilted streamers,” designating them as streamers because they extend equatorward a few degrees in latitude. Similar tilted streamers were identified in Zesta et al. (2006) and shown to map to the tail as primarily radial structures that traverse a large radial extent of the tail, consistent with them being streamers associated with earthward moving tail flow bursts.

As the streamer tilted equatorward toward the diffuse emission band along the equatorward boundary of the oval [this band likely being proton aurora due to precipitation of particles from the partial ring current (e.g. Zou et al. 2009)], onset occurred at 0511:21 UT (identified in the 0511:54 panel of Fig. 1). The onset initiated just to the west of, and ~ 1° of equatorward of, the eastern edge of the tilted streamer. The onset activity then extended westward, consistent with the expected azimuthal drift of low-entropy plasma brought earthward by the streamer associated flow burst, and then expanded poleward.

**Fig. 3:**
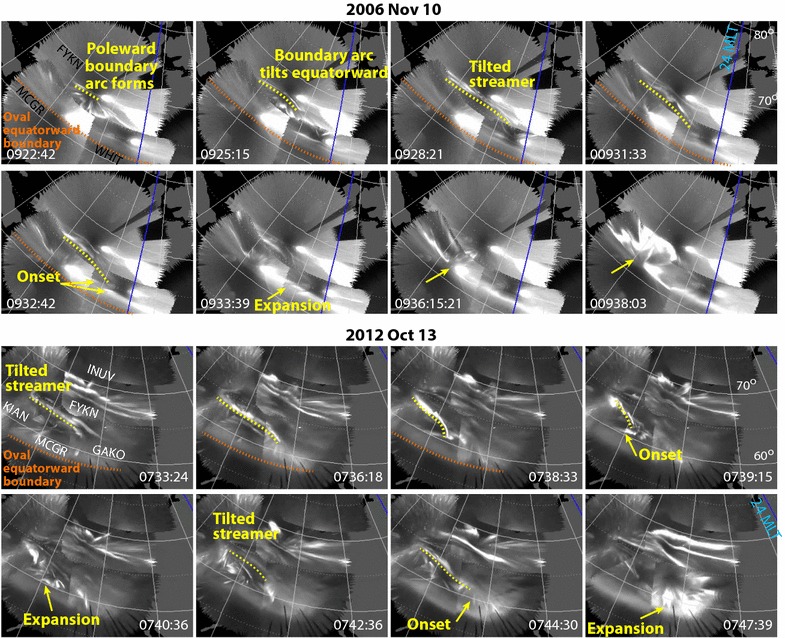
Examples of events on 10 November 2006 and 13 October 2012 in the same format as Fig. 1 that are consistent with onset triggering by tilted streamers that are not as ideal as the event in Fig. 1

Figure 3 shows examples of events that are consistent with onset triggering by tilted streamers that are not as ideal as the event in Fig. 1. In the 10 November 2006 example (auroral movie for this event, with images every 3 s, is given in Additional file 4), a relatively short arc first formed along the auroral poleward boundary (a “poleward boundary intensification”). The arc then tilted equatorward as a tilted streamer leading to onset at 0932 UT just equatorward of the eastern edge of the tilted streamer as the streamers edge reached ~ 1° poleward of the onset location. While the onset was just to the east of the bright light of the moon (which shows in the images from all three ASIs used for the ASI mosaic), the streamer and the onset can be clearly seen.

The events in Fig. 3 on 13 October 2012 (3 s auroral movie in Additional file 5) are the closest together in time of our identified onsets. The activity following the 0739 UT onset was localized in longitude to < 1 h of MLT and was followed by the 0744 UT onset that expanded after the activity for the 0739 UT onset had faded away. While the auroral activity with the first onset has all the characteristics of a substorm and expanded poleward by a substantial ~ 3° in latitude, it did not reach the auroral poleward boundary and would thus be formally classified as a pseudo-breakup. The streamer identified prior to the first onset tilted equatorward to almost the onset location as can be seen in 0738:33 image in Fig. 3. A new tilted streamer first became visible at ~ 0742 UT just poleward and east of the expansion phase activity of the first onset, and its eastern edge then tilted equatorward to near the location of the second onset.

**Fig. 4:**
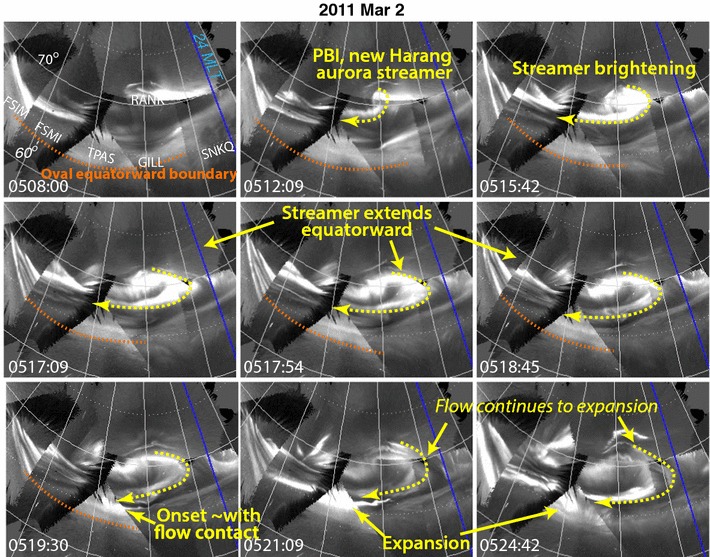
Example of a Harang streamer on 2 March 2011 in the same format as Fig. 1 showing the streamer’s leading edge moving to very near the onset location

Of the 60 onsets in Table 1 that were observed well by the ASIs, we identified 45 for which a tilted streamer appeared to trigger the onset as in the examples in Figs. 1 and 3. For the 14 of the remaining, we identified what we call a “Harang streamer” with its leading edge moving to very near the onset location as illustrated by the example on 2 March 2011 in Fig. 4 and the 3 s movie in Additional file 6. (For one case, onset was clearly observed by we could not determine if it were a tilted or Harang streamers due to tree FOV interference.) In the 2 March 2011 example, a brightening developed at ~ 0510 UT at the westward edge of an arc along the auroral poleward boundary. This PBI quickly developed a Harang reversal shaped streamer. This Harang streamer then expanded with time with its equatorward portion moving equatorward by ~ 5° in latitude until it became very near the diffuse auroral band in the equatorward portion of the oval and led to a substorm onset. Substorm expansion followed, the expansion phase activity appearing to be continually fed by the new, intruding plasma flowing adjacent to the auroral streamer. (While the expansion phase activity for this event became broad in azimuthal coverage, ~ 3 h in MLT, and persisted for ~ 8–10 min, the activity never reached the auroral poleward boundary, so it would be formally classified as a pseudo-breakup.)

The example in Fig. 4 shows that Harang streamers roughly following the large-scale ionospheric flow pattern around the Harang flow shear (e.g., Gkioulidou et al. 2009) of the duskside convection cell and have been identified by Nishimura et al. (2010a) as a type of streamer that can trigger substorm onsets. Our criteria for distinguishing Harang streamers from tilted streamers were based on visually identifying a turning of the streamer to the west before its apparent triggering of an onset. Despite our categorizing of the triggering streamers as either tilted or Harang, the poleward portion of all our identified streamers moved toward the east as they titled equatorward in their poleward portion. Such a tilting is commonly seen for streamers (Zesta et al. 2002, 2006). It is interesting that we detected a higher percentage of substorm onsets that appear to have been triggering by Harang streamers during HSS storms (19%) than during CME storms (12%), though it is not readily clear whether this difference is significant.

## Summary and conclusions

In this paper, we have tested the Nishimura et al. (2010a) scenario for substorm onset triggering by the flow bursts in the plasma sheet that are often manifested in the ionosphere as auroral streamers. We have taken advantage of the broad coverage of the ionospheric mapping of the plasma sheet offered by the THEMIS ASI network to detect substorm onsets and pre-onset streamers, and chosen substorms during geomagnetic storms because of the brighter than normal auroral emissions that occur during storms. We selected storm main phases (9 CME related and 9 HSS related) having good viewing over North America with the THEMIS ASI array.

We observed a streamer heading to near the substorm onset location for all 60 onsets in Table 1 that we identified and were observed well by the ASIs. This is consistent with substorm onsets being very often triggered by the intrusion of plasma with lower entropy than the surrounding plasma to the onset region. However, we note that our auroral observations do not give conclusive proof that the large majority of substorm onsets are triggered by the streamer scenario, since we are not able to directly observe the intrusion of the lower entropy plasma and thus cannot prove that all the streamers we observed were associated with intrusion of lower entropy plasma to the onset region. Direct tests of the intrusion of new plasma to the onset region can be performed using spacecraft (Xing et al. 2010), though sparse coverage of the plasma sheet by spacecraft that makes opportunities for such a test is very limited. Radar observations can also be used, and can allow for direct detection of the ionospheric mapping of flow bursts to the onset region (Nishimura et al. 2014).

It is important that the poleward portion of all the identified streamers tilted equatorward as they extended eastward, consistent with them being guided by the large-scale flow poleward of the Harang reversal. The majority are what we have referred to here as tilted streamers, where they continue to extend eastward as their eastern tip moves equatorward to near the substorm onset location. However, there were 14 of the 60 cases, identified as Harang streamers, where the streamer discernibly turned toward the west poleward of its reaching to near the onset latitude, indicating flow around the Harang reversal. Whether or not tilted streamers are as common during non-stormtimes is not addressable by the current study.

By selecting substorms during both CME and HSS storms, we have been able to check substorm onset occurrence rates during each type of storm. We observed substantially less substorm onsets for CME storms than for HSS storms, this difference being a factor of four between the four CME events having the smoothest southward IMF Bz during the period of good ASI viewing and the four HSS storms with the best combination of good ASI viewing and fluctuating IMF 9 (Fig. 2). This result is inconsistent with Liou et al. (2017). A plausible reason for this discrepancy is their using only the AL index to identify substorms. Specifically, they identified substorms as sharp drops in the SuperMAG AL index by an average amount of 100 nT that persist for 25 min. It is apparent from the SuperMAG AL index for our CME events in Fig. 2 that there are more such drops that are not associated with identified auroral substorms than drops that are associated. We attribute this to the common stormtime occurrence of aurora streamers, which are associated with substantial ground magnetic depressions that are localized to the longitudes of the streamers. This was found to be very common in the detailed study of the 17 March 2013 storm main phase (Lyons et al. 2016), and streamers without substorms have been found to give very clear substorm-like signatures in ground magnetic field observations (Lyons et al. 2013). It would be interesting to check this possibility for the main phases considered here using the THEMIS ASIs and the longitudinally distributed magnetic field measurements from individual stations available from SuperMAG. Additionally, our results from the four CME events with the most steady, strong southward IMF (Fig. 2) are not consistent with the ~ 2–4 h repetition of substorms that has been suggested for moderate to strong southward IMF conditions. Instead, our results are consistent with the findings of Tsurutani et al. (2003) that substorms occurrence is low during such conditions. Our results also support that inferences of Kim et al. (2008) that substorms occur frequently during HSS period due to the highly fluctuating IMF.

This paper has dealt only with the substorm onset process, and our results give no information on what controls the ensuing expansion phase development. Some or our onsets (10 of 80, as indicated in Table 1) led only to a pseudo breaks, some expanded only to the preexisting poleward boundary of the oval, while others expanded poleward (some far poleward) into the preexisting polar cap. Also, some onsets lead to expansion phase activity that was quite localized in longitude, whereas the expansion phase activity of others traversed several hours of nightside MLT. Additionally, expansion phase activity after onset continued for a wide range of time periods, ranging for several minutes to tens of minutes. Our results suggest that the onset process may be similar for this variety of ensuing substorm expansions, which would make it interesting to consider what controls the substorm expansion characteristics.

## Additional files

**Additional file 1.** Movie of image mosaics every 3 s for the tilted streamer example on 19 February 2012 shown in Fig. 1.

**Additional file 2.** Movie of image mosaics every 1 min for 02-11 UT for the 30 April 2014 CME storm.

**Additional file 3.** Movie of image mosaics every 1 min for 02-11 UT for the 27 April 2008 HSS storm.

**Additional file 4.** Movie of image mosaics every 3 s for the tilted streamer example on 10 November 2006 shown in the upper half of Fig. 3.

**Additional file 5.** Movie of image mosaics every 3 s for the two tilted streamer examples on 13 October 2012 shown in the lower half of Fig. 3.

**Additional file 6.** Movie of image mosaics every 3 s for the Harang streamer example on 2 March 2011 shown in Fig. 4.

## Abbreviations

ASI: all-sky-imagers
CME: coronal mass ejection
FOV: field-of-view
HSS: high-speed solar wind stream
Pdym: solar wind dynamic pressure

## References

[CR1] Akasofu S-I (1964). The development of the auroral substorm. Planet Space Sci.

[CR2] Angelopoulos V (2008). The THEMIS mission. Space Sci Rev.

[CR3] Angelopoulos V, Coroniti FV, Kennel CF, Kivelson MG, Walker RJ, Russell CT, McPherron RL, Sanchez E, Meng C-I, Baumjohann W, Reeves GD, Belian RD, Sato N, Friis-Christensen E, Sutcliffe PR, Yumoto K, Harris T (1996). Multipoint analysis of a bursty bulk flow event on April 11, 1985. J Geophys Res.

[CR4] Donovan EF, Mende S, Jackel B, Syrjäsuo M, Meurant M, Voronkov I, Angelopoulos V, Connors M (2006) The azimuthal evolution of the substorm expansive phase onset aurora. In: Syrjäsuo M, Donovan E (eds) Proceedings of international conference on Substorms-8, University of Calgary, Alberta, Canada, pp 55–60

[CR5] Dubyagin S, Sergeev V, Apatenkov S, Angelopoulos V, Nakamura R, McFadden J, Larson D, Bonnell J (2010). Pressure and entropy changes in the flow-braking region during magnetic field dipolarization. J Geophys Res.

[CR6] Frey HU (2010). Comment on “Substorm triggering by new plasma intrusion: THEMIS all-sky imager observations” by Y. Nishimura et al. J Geophys Res Space Phys.

[CR7] Gallardo-Lacourt B, Nishimura Y, Lyons LR, Ruohoniemi JM, Donovan E, Angelopoulos V, McWilliams KA, Nishitani N (2014). Ionospheric flow structures associated with auroral beading at substorm auroral onset. J Geophys Res Space Phys.

[CR8] Gallardo-Lacourt B, Nishimura Y, Lyons LR, Zou S, Angelopoulos V, Donovan E, McWilliams KA, Ruohoniemi JM, Nishitani N (2014). Coordinated SuperDARN THEMIS ASI observations of mesoscale flow bursts associated with auroral streamers. J Geophys Res Space Phys.

[CR9] Gkioulidou M, Wang C-P, Lyons LR, Wolf RA (2009). Formation of the Harang reversal and its dependence on plasma sheet conditions: rice convection model simulations. J Geophys Res.

[CR10] Haerendel G (2011). Six auroral generators: a review. J Geophys Res Space Phys.

[CR11] Henderson MG, Kepko L, Spence HE, Connors M, Sigwarth JB, Frank LA, Singer HJ (2002) The evolution of north–south aligned auroral forms into auroral torch structures: the generation of omega bands and ps6 pulsations via flow bursts. In: Winglee RM (ed) Sixth international conference on substorms. The University of Washington, Seattle, pp 169–174

[CR12] Henderson MG, Reeves GD, Skoug R, Thomsen MF, Denton MH, Mende SB, Immel TJ, Brandt PC, Singer HJ (2006). Magnetospheric and auroral activity during the 18 April 2002 sawtooth event. J Geophys Res Space Phys.

[CR13] Kim H-J, Lee D-Y, Lyons LR (2008). Are repetitive particle injections during high-speed solar wind streams classic substorms?. J Geophys Res.

[CR14] Liou K, Sotirelis T, Richardson I (2017). Substorm occurrence and intensity associated with three types of solar wind structure. J Geophys Res Space Phys.

[CR15] Lyons LR, Nishimura Y, Donovan E, Angelopoulos V (2013). Distinction between auroral substorm onset and traditional ground magnetic onset signatures. J Geophys Res Space Phys.

[CR16] Lyons LR, Gallardo-Lacourt B, Zou S, Weygand JM, Nishimura Y, Li W, Gkioulidou M, Angelopoulos V, Donovan EF, Ruohoniemi JM, Anderson BJ, Shepherd SG, Nishitani N (2016). The 17 March 2013 storm: synergy of observations related to electric field modes and their ionospheric and magnetospheric effects. J Geophys Res Space Phys.

[CR17] Mende SB, Harris SE, Frey HU, Angelopoulos V, Russell CT, Donovan E, Jackel B, Greffen M, Peticolas LM (2008). The THEMIS array of ground-based observatories for the study of auroral substorms. Space Sci Rev.

[CR18] Nakamura R, Baumjohann W, Mouikis C, Kistler LM, Runov A, Volwerk M, Asano Y, Vörös Z, Zhang TL, Klecker B, Rème H, Balogh A (2004). Spatial scale of high-speed flows in the plasma sheet observed by cluster. Geophys Res Lett.

[CR19] Nishimura Y, Lyons L, Zou S, Angelopoulos V, Mende S (2010). Substorm triggering by new plasma intrusion: THEMIS all-sky imager observations. J Geophys Res.

[CR20] Nishimura Y, Lyons LR, Zou S, Angelopoulos V, Mende SB (2010). Reply to comment by Harald U. Frey on “Substorm triggering by new plasma intrusion: THEMIS all-sky imager observations”. J Geophys Res.

[CR21] Nishimura Y, Lyons LR, Kikuchi T, Angelopoulos V, Donovan EF, Mende SB, Chi PJ, Nagatsuma T (2013). Reply to comment by Rae et al. on “Formation of substorm Pi2: a coherent response to auroral streamers and currents”. J Geophys Res Space Phys.

[CR22] Nishimura Y, Lyons LR, Nicolls MJ, Hampton DL, Michell RG, Samara M, Bristow WA, Donovan EF, Spanswick E, Angelopoulos V, Mende SB (2014). Coordinated ionospheric observations indicating coupling between preonset flow bursts and waves that lead to substorm onset. J Geophys Res Space Phys.

[CR23] Oguti T (1973). Hydrogen emission and electron aurora at the onset of the auroral breakup. J Geophys Res.

[CR24] Panov EV, Nakamura R, Baumjohann W, Angelopoulos V, Petrukovich AA, Retinò A, Volwerk M, Takada T, Glassmeier K-H, McFadden JP, Larson D (2010). Multiple overshoot and rebound of a bursty bulk flow. Geophys Res Lett.

[CR25] Pitkänen T, Aikio AT, Amm O, Kauristie K, Nilsson H, Kaila KU (2011). EISCAT-Cluster observations of quiet-time near-Earth magnetotail fast flows and their signatures in the ionosphere. Ann Geophys.

[CR26] Pontius DH, Wolf RA (1990). Transient flux tubes in the terrestrial magnetosphere. Geophys Res Lett.

[CR27] Rae IJ, Murphy KR, Miles DM, Watt CEJ, Mann IR (2013). Comment on “Formation of substorm Pi2: a coherent response to auroral streamers and currents” by Y. Nishimura et al. J Geophys Res Space Phys.

[CR28] Rostoker G, Lui ATY, Anger CD, Murphree JS (1987). North–south structures in the midnight sector auroras as viewed by the Viking imager. Geophys Res Lett.

[CR29] Sakaguchi K, Shiokawa K, Ieda A, Nomura R, Nakajima A, Greffen M, Donovan E, Mann IR, Kim H, Lessard M (2009). Fine structures and dynamics in auroral initial brightening at substorm onsets. Ann Geophys.

[CR30] Samson JC, Lyons LR, Newell PT, Creutzberg F, Xu B (1992). Proton aurora and substorm intensifications. Geophys Res Lett.

[CR31] Sergeev VA, Angelopoulos V, Gosling JT, Cattell CA, Russell CT (1996). Detection of localized, plasma-depleted flux tubes or bubbles in the midtail plasma sheet. J Geophys Res Space Phys.

[CR32] Sergeev VA, Liou K, Meng C-I, Newell PT, Brittnacher M, Parks G, Reeves GD (1999). Development of auroral streamers in association with localized impulsive injections to the inner magnetotail. Geophys Res Lett.

[CR33] Sergeev VA, Sauvaud J-A, Popescu D, Kovrazhkin RA, Liou K, Newell PT, Brittnacher M, Parks G, Nakamura R, Mukai T, Reeves GD (2000). Multiple-spacecraft observation of a narrow transient plasma jet in the Earth’s plasma sheet. Geophys Res Lett.

[CR34] Sergeev V, Nishimura Y, Kubyshkina M, Angelopoulos V, Nakamura R, Singer H (2012). Magnetospheric location of the equatorward prebreakup arc. J Geophys Res.

[CR35] Tsurutani BT, Gonzalez WD (1987). The cause of high-intensity long-duration continuous AE activity (HILDCAAS)—interplanetary Alfven wave trains. Planet Space Sci.

[CR36] Tsurutani BT, Zhou X-Y, Gonzalez WD, Sharma AS, Kamide Y, Lakhina GS (2003). A lack of substorm expansion phases during magnetic storms induced by magnetic clouds. Disturbances in geospace. The storm-substorm relationship.

[CR37] Wolf RA, Chen CX, Toffoletto FR (2012). Thin filament simulations for Earth’s plasma sheet: interchange oscillations. J Geophys Res Space Phys.

[CR38] Xing X, Lyons L, Nishimura Y, Angelopoulos V, Larson D, Carlson C, Bonnell J, Auster U (2010). Substorm onset by new plasma intrusion: THEMIS spacecraft observations. J Geophys Res Space Phys.

[CR39] Yang J, Toffoletto FR, Wolf RA, Sazykin S (2011). RCM-E simulation of ion acceleration during an idealized plasma sheet bubble injection. J Geophys Res.

[CR40] Yang J, Toffoletto FR, Wolf RA (2014). RCM-E simulation of a thin arc preceded by a north-south-aligned auroral streamer. Geophys Res Lett.

[CR41] Zesta E, Lyons L, Donovan E (2000). The auroral signature of earthward flow bursts observed in the magnetotail. Geophys Res Lett.

[CR42] Zesta E, Donovan E, Lyons L, Enno G, Murphree J, Cogger L (2002). Two-dimensional structure of auroral poleward boundary intensifications. J Geophys Res Space Phys.

[CR43] Zesta E, Lyons L, Wang C-P, Donovan E, Frey H, Nagai T (2006). Auroral poleward boundary intensifications (PBIs): their two-dimensional structure and associated dynamics in the plasma sheet. J Geophys Res Space Phys.

[CR44] Zou S, Lyons LR, Nicolls MJ, Heinselman CJ, Mende SB (2009). Nightside ionospheric electrodynamics associated with substorms: PFISR and THEMIS ASI observations. J Geophys Res Space Phys.

